# Unveiling *Wolbachia* transcriptomic signature in the arboviral vector *Aedes aegypti*

**DOI:** 10.3389/fcimb.2025.1538459

**Published:** 2025-04-28

**Authors:** Sebastián Mejías, Natalia E. Jiménez, Carlos Conca, J. Cristian Salgado, Ziomara P. Gerdtzen

**Affiliations:** ^1^ Center for Biotechnology and Bioengineering (CeBiB), University of Chile, Santiago, Santiago, Chile; ^2^ Millennium Nucleus Marine Agronomy of Seaweed Holobionts (MASH), Puerto Montt, Chile; ^3^ Institute for Biological and Medical Engineering, Pontificia Universidad Católica de Chile, Santiago, Santiago Metropolitan Region (RM), Chile; ^4^ Department of Chemical and Bioprocess Engineering, Pontificia Universidad Católica de Chile, Santiago, Santiago Metropolitan Region (RM), Chile; ^5^ Center for Mathematical Modeling, (CMM) (UMI CNRS 2807), Department of Mathematical Engineering, University of Chile, Santiago, Chile; ^6^ Laboratory of Process Modeling and Distributed Computing, Department of Chemical Engineering, Biotechnology and Materials, University of Chile, Santiago, Chile; ^7^ Mammalian Cell Culture Laboratory, Department of Chemical Engineering, Biotechnology and Materials, University of Chile, Santiago, Chile; ^8^ Center of Interventional Medicine for Precision and Advanced Cellular Therapy (IMPACT), Santiago, Chile

**Keywords:** *Aedes aegypti*, *Wolbachia*, arboviral control, transcriptomics, symbiosis

## Abstract

**Introduction:**

The mosquito *Aedes aegypti* is the main vector of arboviral diseases such as dengue and imposes a global health burden. A promising control strategy is to infect *A. aegypti* populations with *Wolbachia*, a genus of intracellular bacteria capable of blocking arboviral infections. Enhancing and preserving the efficacy of this method will depend on a solid mechanistic knowledge of the *A. aegypti-Wolbachia* symbiosis. By identifying differences between *Wolbachia*-infected and uninfected *A. aegypti*, previous transcriptomic studies proposed a wide range of symbiotic interactions, but a systematic identification of consistent effects across datasets is still missing.

**Methods:**

To identify *A. aegypti* genes and functions consistently affected by *Wolbachia*, we performed differential expression and functional enrichment analysis on published transcriptomic datasets, followed by a meta-analysis of the obtained *p-values* using the maxP method. Six datasets were retrieved from Gene Expression Omnibus, Sequence Read Archive and ArrayExpress (last searched in July 2024, considering lack of replication as the exclusion criteria). After discarding one dataset from *w*AlbB-infected cell line due to poor mapping to the *A. aegypti* genome, the data comprised adult female *A. aegypti* heads, muscles, carcasses, midguts and bodies, and Wolbachia strains wMel and wMelPop.

**Results and Discussion:**

Meta-analysis revealed 10 and 21 consistently down- and upregulated host genes, some of which have escaped the focus of previous research, including the consistently downregulated exonuclease *AAEL009650* which has a pro-dengue virus homolog in *Drosophila*. At the function level, we found consistent upregulation of electron transport chain (ETC), carbohydrate transport and serine-type peptidase activity and inhibition, and downregulation of DNA replication. ETC upregulation suggests an alternative mechanism for Wolbachia’s induction of antiviral oxidative stress, previously attributed to dual- and NADPH-oxidases which here showed downregulation or no regulation. Through analysis of previously published datasets, this work identifies promising molecular and functional targets for future studies aimed at elucidating the most fundamental mechanisms of the *A. aegypti–Wolbachia* symbiosis.

## 1 Introduction

Arboviruses (arthropod-borne viruses) such as dengue, Zika and chikungunya viruses (DENV, ZIKV and CHIKV, respectively) impose serious morbidity and mortality burdens worldwide (Flores and O’Neill, 2018; Jones et al., 2020; Yen and Failloux, 2020; Ogunlade et al., 2021). The primary arboviral vector, *Aedes aegypti*, has expanded its geographic range and is now prevalent in Africa, Europe, Asia, Oceania and the Americas (Kraemer et al., 2019; Jones et al., 2020; Ogunlade et al., 2021; Laporta et al., 2023). This, coupled with a general lack of effective broad-spectrum vaccines and the limitations of insecticide-based vector control, has driven the development of new strategies against arboviral diseases (Flores and O’Neill, 2018; Ritchie et al., 2018; Yen and Failloux, 2020; Edenborough et al., 2021). A recent control method centers around *Wolbachia*, a genus of obligate intracellular bacteria naturally occurring in nearly 40% of all arthropod species (Zug and Hammerstein, 2012). *Wolbachia* transfection into the mosquito *A. aegypti* reduces vectorial capacity for arboviruses (pathogen blocking, PB), and induces embryo lethality when infected males mate with uninfected females (cytoplasmic incompatibility, CI), conferring a reproductive advantage to infected females (Raquin et al., 2015; Dainty et al., 2021; Sigle et al., 2022). These phenotypes have motivated controlled releases of transfected mosquitoes to replace wild *A. aegypti* populations, successfully reducing dengue disease in Indonesia (Utarini et al., 2021), Australia (Ryan et al., 2020) and Brazil (Pinto et al., 2021).

Despite current success, there are critical challenges regarding this strategy, such as optimizing the introduction and spread of *Wolbachia* under adverse selective pressures (Hoffmann and Turelli, 2013) and avoiding loss of desired phenotypes over time (Ritchie et al., 2018). Solution of these problems will depend on the mechanistic understanding of the *A. aegypti*-*Wolbachia* symbiosis (Caragata et al., 2013; Lindsey et al., 2018; Sigle et al., 2022). Transcriptomic data allows a comprehensive characterization of the molecular players of symbiosis, as well as the biological processes implicated (Chaston and Douglas, 2012). Consequently, comparative microarray and RNA-Seq analyses of *Wolbachia*-infected versus uninfected *A. aegypti* have made notable progress in determining the impacts of this bacterium on immune response, metabolism, redox homeostasis and behavior, identifying specific mediating host genes (Kambris et al., 2009; Pan et al., 2012; Rancès et al., 2012; Ye et al., 2013; Mao et al., 2022; Boehm et al., 2023; Wimalasiri-Yapa et al., 2023).

Despite these key contributions, the extent to which the diverse reported effects of *Wolbachia* are shared across conditions such as bacterial strain or host tissue and age is still unclear. Identifying genes and functions consistently affected by *Wolbachia* across studies is key, as these likely represent fundamental points of interaction that would be relevant for all instances of *Wolbachia*-based arboviral control strategies. Although this task has been addressed in the context of specific multifactorial experiments (Rancès et al., 2012; Wimalasiri-Yapa et al., 2023), no study has been dedicated to conciliate all available transcriptomic data accumulated over the last two decades in this system.

A meta-analysis provides a systematic way for finding consistent effects in differential gene expression, increasing statistical power for detection of genes with mild but consistent effects across datasets (Li and Ghosh, 2014; Yoon et al., 2021), which may be overlooked in individual studies. In particular, a meta-analysis based on *de novo* analysis of raw data allows for the extraction of previously unrecognized insights from individual studies, which despite producing genome-wide data have focused on specific traits, such as differentially methylated genes (Ye et al., 2013), long non-coding RNAs (Mao et al., 2022), immune genes (Kambris et al., 2009), or upregulated genes (Rancès et al., 2012). Bioinformatic resources such as Gene Ontology evolve in time, and this significantly affects interpretations of genomic screenings based on these resources (Tomczak et al., 2018), further highlighting the value of a *de novo* analysis of raw data published decades ago. Overall, a *de novo* meta-analysis can unveil consistent and condition-specific points of interaction between *Wolbachia* and *A. aegypti*, highlighting concrete molecular targets for future functional investigations.

In this work, we present a *de novo* meta-analysis of published transcriptomic datasets from *Wolbachia*-infected and uninfected *A. aegypti*, to unveil a transcriptomic signature of *Wolbachia* in this mosquito. By addressing limitations in previous analyses and synthesizing results, we aim to contribute significantly to the broader understanding of the *A. aegypti*-*Wolbachia* symbiosis and relevant phenotypes for arboviral control.

## 2 Materials and methods

### 2.1 Data collection

Comparative genome-wide microarray and RNA-Seq datasets comprising *Wolbachia*-infected and uninfected *A. aegypti* conditions were retrieved from Gene Expression Omnibus (GEO) (Edgar et al., 2002), Sequence Read Archive (SRA) (Leinonen et al., 2011) and ArrayExpress (Parkinson et al., 2007), all last searched in July 2024 using the keywords ‘wolbachia’ and ‘aedes aegypti’ in conjunction. Lack of biological replication or *Wolbachia*-uninfected controls were considered as the only exclusion criteria, as the first one would impede an assessment of intra-condition variability for differential expression analysis, while the second one would preclude an evaluation of *Wolbachia*’s effect altogether.

Outcomes extracted from RNA-Seq and microarray datasets were, respectively, sequencing reads and intensity values for two-channel spots competitively hybridized to cDNA from contrasting conditions (*Wolbachia*-infected *vs* uninfected). All outcomes were considered for analysis, except those corresponding to ZIKV-infected *A. aegypti* from the SRA accession PRJNA949154 (Boehm et al., 2023), as our work was focused on baseline effects of *Wolbachia*’s infection in *A. aegypti*. SRA accessions PRJNA722598 and PRJNA789930 (both corresponding to the study from (Mao et al., 2022) and thus considered here as a single dataset) were excluded *a posteriori* due to low mapping rates to the *A. aegypti* genome. See **Supplementary Table 1** for a summary of included and excluded datasets, and **Supplementary Table 2** for mapping statistics of RNA-Seq data.

### 2.2 Microarray data analysis

All analyses were performed using R v4.2.3 (R Core Team, 2024) with the limma v3.54.2 package (Ritchie et al., 2015), with default parameters and standard methods unless stated otherwise. To prevent unreliable microarray spots from affecting normalization steps, weights of outlier and quality flagged spots were set to zero. After background correction, two-channel intensity data was combined into log-ratio values, followed by within- and between-array normalization. Control spots were removed and normalized log-ratios from duplicated spots were averaged before differential expression analysis, contrasting each *Wolbachia*-infected condition to its equivalent uninfected condition. Principal component analysis (PCA) and heatmaps were based on normalized log-intensities for each channel, and visualized using R packages ggplot2 v3.5.1 (Wickham, 2016) and pheatmap v1.0.12 (Kolde, 2018), respectively.

To identify functional classes overrepresented among up- or downregulated genes from each dataset, each gene list was sorted in decreasing order according to its logarithmic fold-change and subjected to Gene Set Enrichment Analysis (GSEA) (Subramanian et al., 2005), using the R package fgsea v1.30.0 (Korotkevich et al., 2019) against Gene Ontology functional terms (database version 2024-01) (Harris et al., 2004). GSEA was preferred over other methods as it does not require applying arbitrary significance or size-effect cutoffs on differential expression results, which may hinder the identification of functional effects reflected by moderate but coordinated expression changes of several genes (Subramanian et al., 2005). GSEA overcomes this limitation by considering the complete results of a differential expression analysis, quantifying the extent to which the genes from each functional set are enriched towards the up- or downregulated genes via a Normalized Enrichment Score (positive/negative scores reflect up/downregulation, respectively) and its *p*-value (Subramanian et al., 2005). This approach is particularly valuable as *Wolbachia* is restricted to some host tissues and its effects are to a great extent tissue-specific (Baião et al., 2019; Boehm et al., 2023), so that local transcriptomic signals may be diluted in whole body RNA samples. *p*-values were Benjamini-Hochberg adjusted to control the False Discovery Rate (Benjamini and Hochberg, 1995), applying a threshold of 0.05. Enrichment plots were generated for the 10 up/downregulated GO terms with largest Normalized Enrichment Score in magnitude, using ggplot2 (Wickham, 2016).

### 2.3 RNA-seq data analysis

Quality of raw *A. aegypti* reads was assessed with FastQC v0.11.9 (Andrews, 2010). Trimmomatic v0.38 (Bolger et al., 2014) was used to trim adapter and low quality sequences, using a sliding window approach. STAR v2.7.8a (Dobin et al., 2013) was used to align trimmed reads to the AaegL5 reference genome, with splicing event information obtained from NCBI RefSeq *A. aegypti* Annotation Release 101 (O’Leary et al., 2016; Matthews et al., 2018). Aligned reads were counted at gene level using featureCounts v2.0.1 (Liao et al., 2014) and differential expression analyses were performed using DESeq2 v1.34.0 (Love et al., 2014) in R, contrasting each *Wolbachia*-infected condition to its equivalent uninfected condition. Principal component analysis and heatmaps were based on gene counts normalized via the Variance Stabilizing Transformation (VST) from DESeq2 (Love et al., 2014). GSEA was performed and visualized as previously described.

### 2.4 Meta-analysis

To search for *A. aegypti* genes and functions consistently affected by *Wolbachia* infection, we used the maxP method (Wilkinson, 1951) implemented in the metap R package (Dewey, 2024) to combine *p*-values derived from individual differential expression and GSEA tests. maxP is appropriate for finding traits with non-null effects in all studies from a collection. Mathematically, maxP tests the conjunction null hypothesis $H_{0}:\;{\cap^{}}_{k}\left\{\theta_{gk}=0\right\}$ versus $H_{a}:\;{\cup^{}}_{k}\left\{\theta_{gk}\neq 0\right\}$ , where $\theta_{gk}$ is the effect of a given trait *g* (genes or gene sets) in the study *k*. This is achieved using the maximum *p*-value among all tests as the summary test statistic, which follows a Beta distribution with parameters α=K (the number of studies) and β=1 (Chang et al., 2013; Siangphoe and Archer, 2017). Combined *p*-values (*i.e.* those resulting from testing the conjunction null hypothesis) were adjusted with the Benjamini-Hochberg method to control the False Discovery Rate (Benjamini and Hochberg, 1995), applying a cutoff of 0.05 for genes and 0.01 for GO terms. Genes and functions with missing *p*-values in any comparison (*e.g.* genes not included in a microarray dataset) were excluded from meta-analysis. Functional gene annotations were retrieved from VectorBase (Giraldo-Calderón et al., 2015), InterPro (Jones et al., 2014) and Gene Ontology (Harris et al., 2004). *A. aegypti* genes belonging to the Toll, IMD and JAK/STAT pathways were defined using the taxonomy-based filtering of pathways from Kyoto Encyclopedia of Genes and Genomes (Kanehisa et al., 2023).

## 3 Results

### 3.1 Overview of datasets

Six datasets published between 2009 and 2023 were retrieved (Kambris et al., 2009; Rancès et al., 2012; Ye et al., 2013; Mao et al., 2022; Boehm et al., 2023; Wimalasiri-Yapa et al., 2023). The dataset composed of SRA accessions PRJNA722598 and PRJNA789930 was discarded *a posteriori* due to low mapping to the *A. aegypti* genome (all samples had <36% reads mapped, **Supplementary Table 2**). Remaining datasets comprised *Wolbachia* strains *w*Mel and *w*MelPop and five kinds of host tissue: whole body, head, muscle, carcass and midgut. All samples were female adults, ranging from 2 to 15 days post-eclosion (**Table 1**).

**Table 1:** Summary of analyzed transcriptomic datasets.

Accession no. ^a^	Study	Assay	Background condition	Experimental groups ^b^	Comparison
PRJNA118709	Kambris et al. (2009)	Array	2 and 15 days-old female whole bodies from laboratory lines	A. Line PGYP1 B. Line PGYP1.tet	A vs B
E-MEXP-2931	Rancès et al. (2012)	Array	8 days-old female whole bodies from laboratory lines	A. Line PGYP1 B. Line PGYP1.tet C. Line MGYP2 D. Line MGYP2.tet	A vs B C vs D
E-MEXP-2907	Ye et al. (2013)	Array	15 days-old female tissues from laboratory lines	A. Line PGYP1, head B. Line PGYP1.tet, head C. Line PGYP1, muscle D. Line PGYP1.tet, muscle	A vs B C vs D
PRJNA867516	Wimalasiri-Yapa et al. (2023)	RNASeq	4 days-old female whole bodies from natural populations with given *w*Mel release histories	A. *w*Mel released in 2011 B. *w*Mel released in 2013/14 C. *w*Mel released in 2017 D. No release	A vs D B vs D C vs D
PRJNA949154	Boehm et al. (2023)	RNASeq	7 to 13 days-old female tissues from laboratory lines, at given days post blood feeding (dpf)	A. Line COL.wMel, carcass, 4 dpf B. Line COL.tet, carcass, 4 dpf C. Line COL.wMel, carcass, 7 dpf D. Line COL.tet, carcass, 7 dpf E. Line COL.wMel, midgut, 4 dpf F. Line COL.tet, midgut, 4 dpf G. Line COL.wMel, midgut, 7 dpf H. Line COL.tet, midgut, 7 dpf	A vs B C vs D E vs F G vs H

a E-MEXP prefix corresponds to ArrayExpress accession numbers, while PRJNA prefix corresponds to SRA and GEO accession numbers.

b Lines PGYP1 and PGYP1.tet: *w*MelPop-transinfected *A. aegypti* and its tetracycline cleared counterpart (Kambris et al., 2009; Rancès et al., 2012; Ye et al., 2013). Lines MGYP2 and MGYP2.tet: *w*Mel-transinfected *A. aegypti* and its tetracycline cleared counterpart (Rancès et al., 2012). Lines COL.wMel and COL.tet: *w*Mel-transinfected Colombian *A. aegypti* and its tetracycline cleared counterpart (Boehm et al., 2023).

Datasets were analyzed *de novo*, yielding reliable results according to intermediate step quality assessments. In particular, >90% reads mapped to the *A. aegypti* reference genome in each RNA-Seq sample (**Supplementary Table 2**) and microarray datasets show consistency between expected and observed M values (logarithmic fold-change between red and green intensities) and A values (average of logarithmic red and green intensities) for control spots (depicted by MA-plots in **Supplementary Figure 1**). A description of each original study as well as new results obtained in this work is provided in the following sections.

### 3.2 Kambris et al. (2009)

Kambris et al. (2009) sought to examine the mechanisms behind the life-shortening phenotype induced by *Wolbachia* in *A. aegypti*, comparing whole-body transcriptome of female *w*MelPop-infected *A. aegypti* (line PGYP1) versus tetracycline-treated PGYP1 cells to remove *Wolbachia* infection (line PGYP1.tet). Four two-channel Agilent microarray slides were competitively hybridized to PGYP1 and PGYP1.tet cDNA samples, each sample derived from an independent pool of 2- and 15-days-old mosquitoes. No functional enrichment analysis of obtained differentially expressed genes (DEGs) was performed (Kambris et al., 2009).

**Figure 1:**
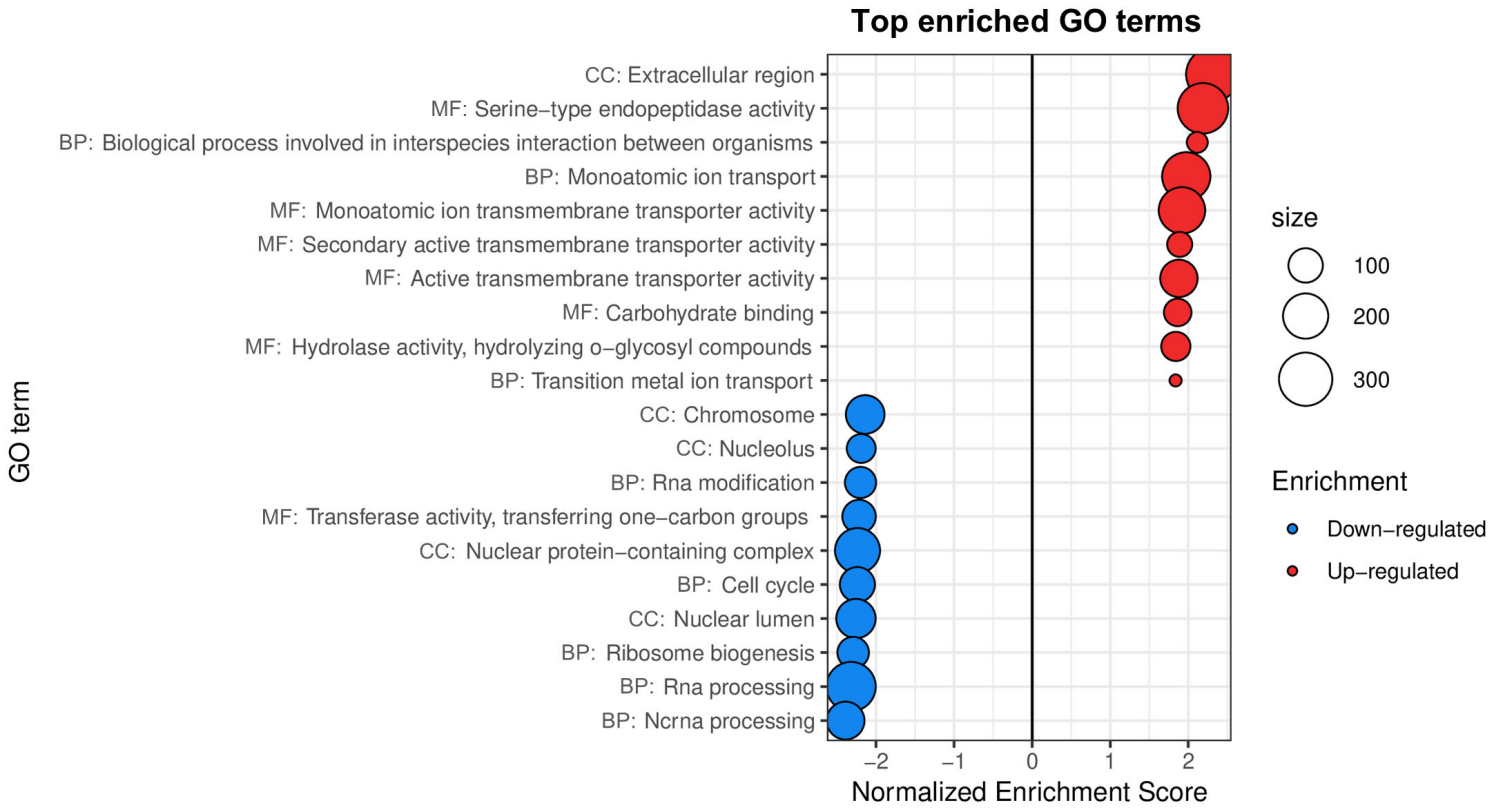
Gene sets impacted by *w*MelPop on female *A. aegypti* whole bodies, assessed from the analysis of the dataset in Kambris et al. (2009). The figure displays the top GO terms ranked by Normalized Enrichment Score, reflecting the overrepresentation of each term among genes upregulated (positive scores) or downregulated (negative scores) by *w*MelPop. BP, Biological Process; CC, Cellular Component; MF, Molecular Function.

Our analysis yielded new insights into the transcriptomic effect of *w*MelPop on *A. aegypti* (**Figure 1**). Infection status was not the main source of variation in this dataset (**Supplementary Figure 2A**). Indeed, PCA showed that the first principal component (58% of total variance) correlates with slides rather than infection status, suggesting a residual batch effect not removed in the between-array normalization step. Nevertheless, an overall effect of *w*MelPop on host gene expression was still distinguishable by the second principal component, which comprised 14% of the total variance, allowing the identification of 13 up- and 30 downregulated GO terms (**Supplementary Table 3**).

Results were widely consistent with the immune activation previously reported by Kambris et al. (2009), as top 10 upregulated GO terms Serine-type endopeptidase activity, Extracellular region and Carbohydrate binding (**Figure 1**) were driven by the overexpression of CLIP-domain serine proteases (CLIPs), pattern recognition receptors, prophenoloxidases and defensins (**Supplementary Table 3**). Our analysis further revealed effects of *w*MelPop in host transport processes (**Figure 1**) not reported in the original publication, suggesting an impact of *w*MelPop on amino acid homeostasis (including four amino acid transporters), transition metal homeostasis (including transporters of iron, zinc and copper), intercompartmental pH balance (comprising four vacuolar ATP synthase subunits) and neuronal signaling (including neurotransmitter receptors and ion channels) (**Supplementary Table 3**).

Top 10 downregulated GO terms (**Figure 1**) suggested that *w*MelPop modulates RNA post-transcriptional modification patterns, with underexpression of putative ribonucleases, splicing factors, tRNA-methyltransferase, a nuclear cap-binding protein and a pre-mRNA cleavage factor involved in mRNA polyadenylation (**Supplementary Table 3**). The results also hinted at a coordinated suppression of key elements for cell cycle regulation and DNA metabolism. Indeed, downregulation of GO terms Chromosome, Cell cycle (**Figure 1**) and DNA metabolic process (**Supplementary Table 3**) was driven by the underexpression of histone proteins, DNA damage checkpoint protein, DNA replication licensing factor, DNA methyltransferase, replication factor C subunit, centromere/kinetochore protein, CDK (cyclin-dependent kinase) activating kinase and CDK activating kinase assembly factor, among others (**Supplementary Table 3**).

### 3.3 Rancès et al. (2012)

**Figure 2:**
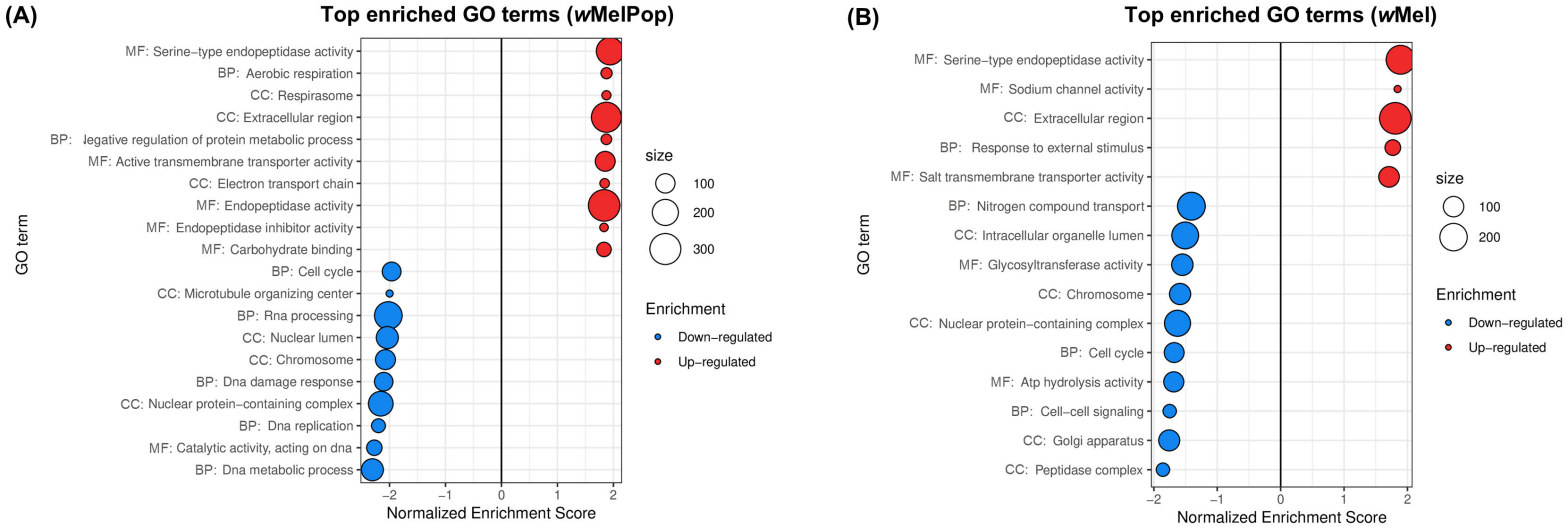
Gene sets impacted by *w*MelPop and wMel on female *A*. *aegypti* whole bodies, assessed from the analysis of the dataset in Rancès et al. (2012). The figure displays the top GO terms ranked by Normalized Enrichment Score, reflecting the overrepresentation of each term among genes upregulated (positive score) or downregulated (negative score) by **(A)** *w*MelPop and **(B)** *w*Mel. BP, Biological Process; CC, Cellular Component; MF, Molecular Function.

Rancès et al. (2012) aimed to identify immune genes involved in *Wolbachia*’s pathogen blocking in *A. aegypti*, comparing whole-body transcriptome of female *w*MelPop- and *w*Mel-infected mosquitoes (lines PGYP1 and MGYP2) versus their tetracycline-treated counterparts (lines PGYP1.tet and MGYP2.tet). 12 two-channel Agilent microarray slides were competitively hybridized to *Wolbachia*-infected and uninfected cDNA samples (6 for PGYP1 *vs* PGYP1.tet and 6 for MGYP2 *vs* MGYP2.tet), each sample derived from an independent pool of twenty 8-days-old mosquitoes. Upregulated genes were subjected to overrepresentation analysis against the distribution of GO categories for the *A. aegypti* genome (Rancès et al., 2012). Our *de novo* analysis of this dataset (**Figure 2**) complemented the identification of upregulated functions and provided identification of new downregulated functions.

Results showed a strong transcriptomic effect of both infection status and *Wolbachia* strain (**Supplementary Figure 2B**), with a stronger overall effect of *w*MelPop than *w*Mel. This was depicted as the separation of infected and uninfected samples according to PC1 (32% of total variance) was more pronounced for *w*MelPop, which is consistent with a larger number of DEGs reported by Rancès et al. (2012) for that strain. In total, 27 up- and 30 downregulated GO terms were found here for *w*MelPop-infected *A. aegypti* and 5 up- and 10 downregulated GO terms for *w*Mel-infected *A. aegypti* (**Supplementary Table 3**).

Our results were consistent with the immune activation reported by Rancès et al. (2012), as top 10 upregulated GO terms Serine-type endopeptidase activity and Extracellular region (**Figures 2A, B**) reflected the overexpression of CLIPs, serine protease inhibitors, transferrin and defensin, as well as prophenoloxidases in the case of *w*MelPop (**Supplementary Table 3**). Upregulation of Electron transport chain and Respirasome (**Figure 2A**) was also consistent with the upregulation of Electron carrier activity reported by the authors.

As in the original analysis by Rancès et al. (2012), we found that *w*MelPop upregulates transmembrane transport (**Figure 2A**), with underlying upregulation of amino acid transporters, synaptic vesicle proteins, glutamate and sugar transporters, vacuolar ATPases and ion channels (**Supplementary Table 3**). However, we found that this broad effect on transport functions is not restricted to *w*MelPop, as *w*Mel upregulated Salt transmembrane transporter activity (**Figure 2B**), comprising amino acid transporters, ion channels, calcium-transporting ATPase and mitochondrial carrier proteins (**Supplementary Table 3**).

Our analysis of downregulated genes suggested disruption of DNA replication and cell cycle by both *w*Mel and *w*MelPop, which was not identified in the original study. Top 10 downregulated terms Cell cycle and Chromosome (**Figures 2A, B**) reflected underexpression of DNA polymerase subunits, DNA repair proteins, DNA helicases, anaphase-promoting complex subunits, M-phase inducer phosphatase and a putative cyclin (**Supplementary Table 3**). For *w*MelPop, downregulation of these terms was further driven by additional cyclins, CDK, CDK activating kinase and a CDK regulatory subunit (**Supplementary Table 3**). *w*MelPop also downregulated RNA processing (**Figure 2A**), with underexpression of ribonucleases, ribonucleoproteins, RNA methyltransferases, splicing factors and cap-binding protein subunits (**Supplementary Table 3**); as well as microtubule-mediated transport (**Figure 2A**), with underexpression of tubulin chains, katanin P80 subunit, gamma-tubulin complex component, kinesin and dynein chains (**Supplementary Table 3**).

### 3.4 Ye et al. (2013)

**Figure 3:**
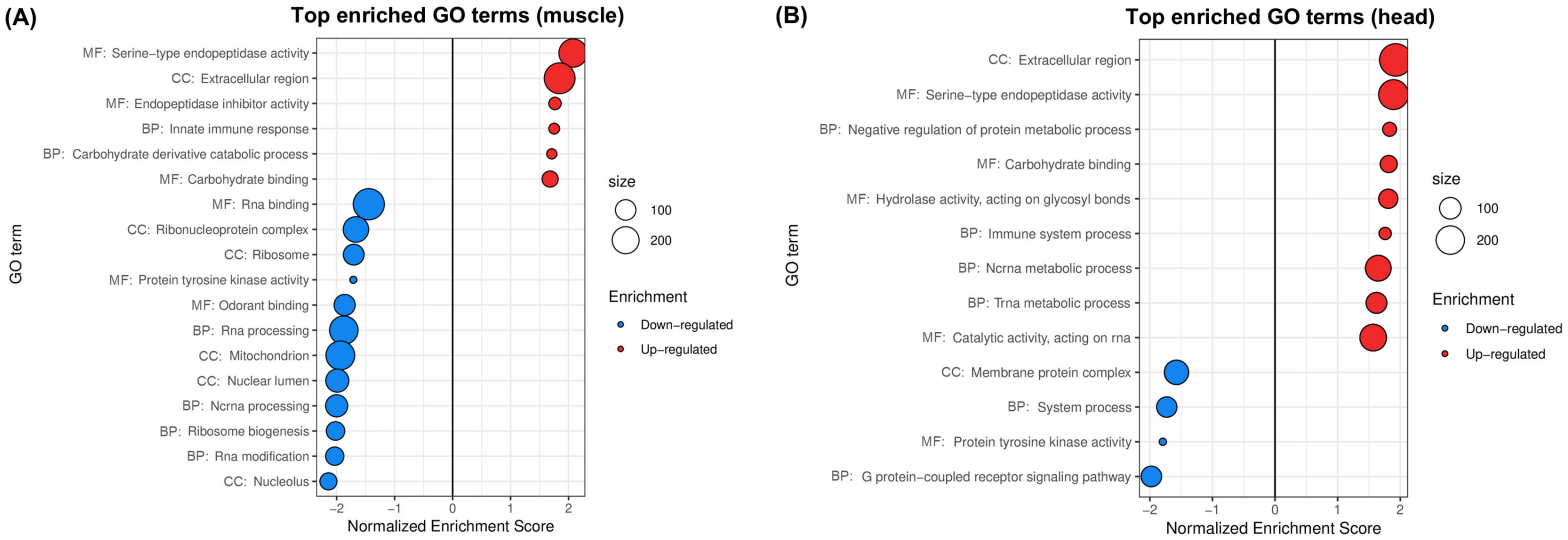
Gene sets impacted by *w*MelPop on female *A*. *aegypti* muscles and heads, assessed from the analysis of the dataset in Ye et al. (2013). The figure displays the top GO terms ranked by Normalized Enrichment Score, reflecting the overrepresentation of each term among genes upregulated (positive score) or downregulated (negative score) by *w*MelPop in **(A)** muscles and **(B)** heads. BP, Biological Process; CC, Cellular Component; MF, Molecular Function.

Ye et al. (2013) characterized the methylome of female *w*MelPop-infected and uninfected *A. aegypti* (lines PGYP1 and PGYP1.tet) heads and muscles, examining its relationship with gene expression changes. 12 two-channel Agilent microarrays were competitively hybridized to PGYP1 and PGYP1.tet cDNA samples (6 for heads and 6 for muscles), each sample derived from a pool of 20 heads or muscle tissues. Differential expression analysis was used to search for correlations between gene methylation and expression across conditions, however, no functional characterization of the genome-wide expression patterns was performed (Ye et al., 2013). We analyzed this dataset to identify the main functional effects of *w*MelPop in *A. aegypti*’s heads and tissues (**Figure 3**).

PCA and hierarchical clustering of transcriptomic profiles (**Supplementary Figure 2C**) showed that tissue was the main factor influencing global gene expression in this dataset (depicted by PC1, comprising 50% of total variance). Still, the impact of *w*MelPop was distinguishable from the PCA, where infected and uninfected samples were segregated by PC2 (12% of total variance). In total, we found 6 up- and 12 downregulated GO terms in *w*MelPop-infected muscles (**Figure 3A**) and 9 up- and 4 downregulated GO terms in *w*MelPop-infected heads (**Figure 3B**).

Infected muscles showed upregulation of Serine-type endopeptidase activity, Extracellular region and Carbohydrate binding, and downregulation of RNA processing (**Figure 3A**), which was consistent with our analyses presented previously for *w*MelPop-infected whole bodies (**Figures 1**, **2A**). Our results also suggested a disruption of aerobic energy metabolism in muscles, with downregulation of the GO term Mitochondrion (**Figure 3A**), driven by the underexpression of NADH dehydrogenases, ATP synthase components, mitochondrial pyruvate carrier and mitochondrial ribosomal proteins, among others (**Supplementary Table 3**).

Infected head tissues exhibited an increased demand for protein synthesis due to *w*MelPop, with upregulation of tRNA metabolism (**Figure 3B**) driven by overexpression of aminoacyl-tRNA synthetases for 13 of the 20 standard amino acids, among others (**Supplementary Table 3**). We also found that *w*MelPop downregulated G protein-coupled receptor (GPCR) signaling pathway in *A. aegypti* heads (**Figure 3B**), including underexpression of G-protein subunits, neuropeptides and several GPCRs including 6 long and short wavelength opsins (**Supplementary Table 3**). Downregulation of these genes may have profound impacts for diverse insect physiological processes regulated by GPCRs, such as reproduction, neurotransmission and stress responses. Particularly, as opsins are key mediators of phototransduction and mosquito light-driven behavior (Zhan et al., 2021; Liu et al., 2022), their downregulation may contribute to *w*MelPop-infected *A. aegypti*’s fitness loss (Ritchie et al., 2015; Allman et al., 2020).

### 3.5 Wimalasiri-Yapa et al. (2023)

**Figure 4:**
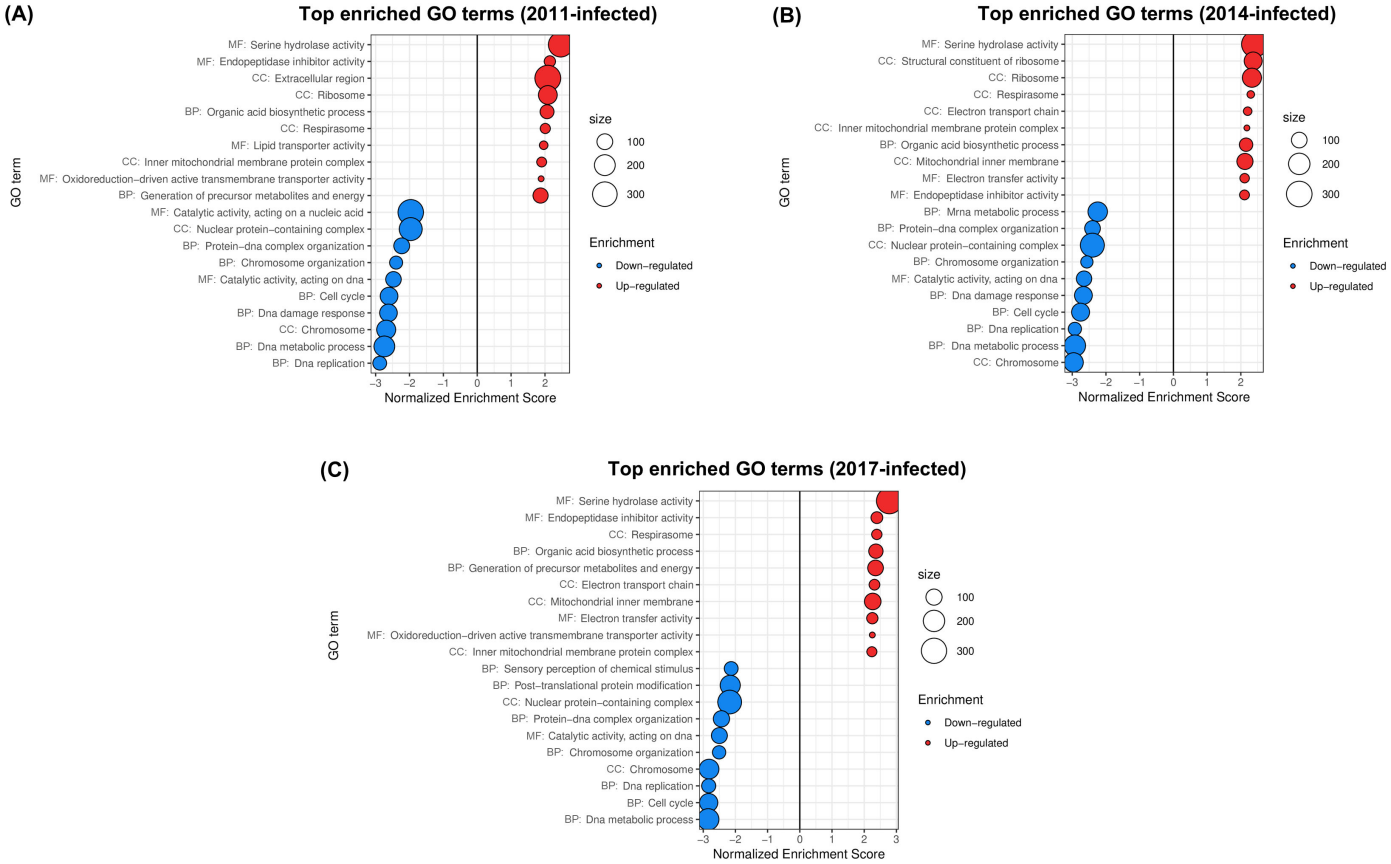
Gene sets impacted by *w*Mel on female *A*. *aegypti* whole bodies from native populations introgressed with *Wolbachia*, assessed from the analysis of the dataset in Wimalasiri-Yapa et al. (2023). The figure displays the top GO terms ranked by Normalized Enrichment Score, reflecting the overrepresentation of each term among genes upregulated (positive score) or downregulated (negative score) by *w*Mel in native populations introgressed in **(A)** 2011, **(B)** 2014 and **(C)** 2017. BP, Biological Process; CC, Cellular Component; MF, Molecular Function.

Wimalasiri-Yapa et al. (2023) studied the temporal progression of *A. aegypti*’s transcriptional response to *Wolbachia* in field conditions, considering Australian mosquito populations that were subjected to *w*Mel releases in 2011, 2013/2014 and 2017 (hereafter, the “2011-, 2014- and 2017-infected populations”) and an uninfected population. Illumina NovaSeq6000 reads were used to compare the whole-body transcriptome of *w*Mel-infected female mosquitoes with each release history versus the uninfected population. The 2011-, 2014- and 2017-infected populations and the uninfected one were represented by 12, 9, 7 and 5 independent samples, respectively, each one consisting in a pool of five 4-days-old females. Enrichment analysis of functional categories were performed on DEG sets (Wimalasiri-Yapa et al., 2023). Our analysis (**Figure 4**) offered complementary insights into the functional changes induced by *w*Mel in wild *A. aegypti* populations.

PCA and hierarchical clustering of transcriptomic profiles (**Supplementary Figure 2D**) grouped uninfected samples together. PC1 (41% of the total variance) segregated uninfected from infected samples, with the greatest distance being between most recent introgression (2017) and the uninfected status. This was consistent with the highest number of DE genes being found for the 2017-infected population in the original publication. The total number of up/downregulated GO terms found was 30/28 for the 2011-infected population, 35/33 for the 2014-infected population and 49/36 for the 2017-infected population (**Supplementary Table 3**).

Upregulation of Serine hydrolase activity and Endopeptidase inhibitor activity in all infected populations (**Figure 4**) was consistent with the results of Wimalasiri-Yapa et al. (2023). Upregulation of Organic acid biosynthetic process (**Figure 4**), driven by overexpression of several genes implicated in fatty acid biosynthesis (**Supplementary Table 3**), was consistent with the enrichment of Fatty acid biosynthetic process reported by the authors for all populations. Our results further indicated enhancement of host aerobic respiration by *w*Mel (**Figure 4**) in all infected populations, which was not identified in the original publication and was driven by upregulation of succinate dehydrogenase, cytochromes, ATP synthase components and NADH dehydrogenases, among others (**Supplementary Table 3**).

GO terms that were among the top 10 downregulated in all comparisons were in concordance with the original analysis of Wimalasiri-Yapa et al. (2023), denoting a disruption of cell cycle and DNA metabolic processes (**Figure 4**), driven by downregulation of cyclins, DNA replication licensing factors, endonucleases, DNA polymerase components, DNA topoisomerase, origin recognition complex subunit, DNA mismatch repair protein and DNA helicase, among others (**Supplementary Table 3**).

### 3.6 Boehm et al. (2023)

**Figure 5:**
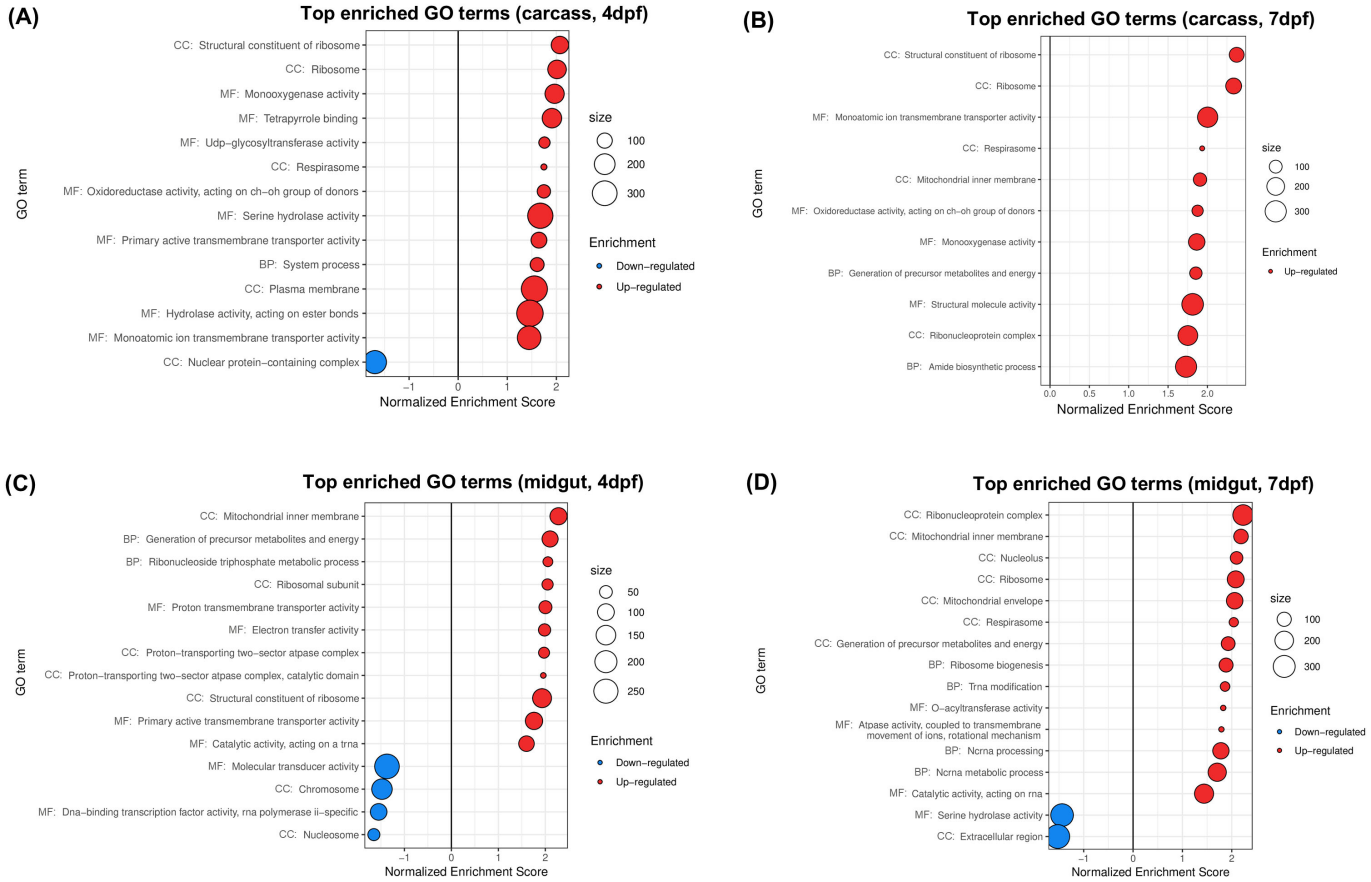
Gene sets impacted by *w*Mel on female *A*. *aegypti* carcasses and midguts at different days post blood feeding (dpf), assessed from the analysis of the dataset in Boehm et al. (2023). The figure displays the top GO terms ranked by Normalized Enrichment Score, reflecting the overrepresentation of each term among genes upregulated (positive score) or downregulated (negative score) by *w*Mel in **(A)** carcasses at 4dpf, **(B)** carcasses at 7dpf, **(C)** midguts at 4dpf and **(D)** midguts at 7dpf. BP, Biological Process; CC, Cellular Component; MF, Molecular Function.

Boehm et al. (2023) studied the transcriptomic effects of *w*Mel on *A. aegypti* carcasses and midguts at different times post-blood feeding and with different status of ZIKV infection. We limit the following description to what concerns the experiments without viral infection. Illumina NovaSeq reads were used to compare the carcass and midgut transcriptomes of *w*Mel-infected and uninfected female mosquitoes at 4 or 7 days post-feeding (dpf). Each combination of time post-feeding, tissue and *Wolbachia* status was represented by 3 biological replicates, each one comprising 30 midguts or carcasses. Reads were subjected to differential expression analysis and GSEA was performed on normalized data (Boehm et al., 2023). In the following we present the results of our own analysis of this dataset (**Figure 5**). PCA and hierarchical biclustering (**Supplementary Figure 2E**) posed tissue as the main grouping factor, separating samples along the PC1, which explained 88% of the total variance. PC2 distinguished infected from uninfected carcasses. Time post feeding appeared as the least impacting factor on global gene expression profiles. The total number of up/downregulated GO terms found was 13/1 for carcass at 4dpf (**Figure 5A**), 11/0 for carcass at 7dpf (**Figure 5B**), 11/4 for midguts at 4 dpf (**Figure 5C**), and 14/2 for midguts at 7 dpf (**Figure 5D**).

Results show upregulation of respirasome (**Figures 5A, B, D**), driven by overexpression of cytochromes c, cytochromes c oxidases and NADH dehydrogenases, among others (**Supplementary Table 3**), consistent with the original publication. Our results went one step further showing a common upregulation of ribosomal components (**Figures 5A, B–D**), with overexpression of several 40S, 60S and mitochondrial ribosomal proteins (**Supplementary Table 3**), representing new functional insights that were not obtained in the original analysis.

Top 10 downregulated genes for carcasses and midguts at 4 dpf denoted suppression of nuclear functions (**Figures 5A, C**), shown by underexpression of genes linked to DNA metabolism, RNA transcription and processing, chromosome maintenance and cell cycle regulation (**Supplementary Table 3**). This was consistent with the downregulation of GO terms Nucleus and Chromosome reported by Boehm et al. (2023) for these conditions. In midguts at 7 dpf (**Figure 5D**) we found downregulation of Serine hydrolase activity, explained by the underexpression of serine-type peptidases, and Extracellular region, explained by underexpression of serine-type peptidases and their inhibitors, thrombospondin, prophenoloxidase and lipases (**Supplementary Table 3**). This result is consistent with the original analysis of Boehm et al. (2023) for midguts at 7 dpf, however, it contrasted with our analysis of all previous datasets, where these GO terms were upregulated (**Supplementary Table 3**).

### 3.7 Consistent transcriptomic effects of *Wolbachia* in *A. aegypti*

To find functions consistently affected by *Wolbachia* on its host, *p*-values derived from individual differential expression and GSEA tests were combined using the maxP approach (see Methods), finding 1,483 and 57 significant genes and GO terms, respectively (**Supplementary Table 4**). We further constrained our focus on genes that: (i) had a consistent direction of differential expression across tests (*i.e.*, logarithmic fold-change always positive or negative), and (ii) were individually significant (*p*<0.05) in at least 8 of the 12 differential expression tests. Genes fulfilling these conditions were called consistently up- or downregulated genes (**Tables 2**, **3**, respectively). We applied a similar criterion for focusing on consistently up- or downregulated functions but, given that no significant GO term was always up- or downregulated, we required at least 10 tests with the same regulation direction (**Table 4**).

**Table 2:** Genes consistently upregulated by *Wolbachia* in *A. aegypti*.

Gene ID	# significant tests (*p*<0.05)	Mean Fold-change^a^	Description	Category
*AAEL001508*	11/12	4.0 ± 1.9	InterPro: Farnesoic acid O-methyltransferase	Metabolism
*AAEL004667*	11/12	3.1 ± 1.5	InterPro: Farnesoic acid O-methyltransferase	
*AAEL000829*	11/12	1.3 ± 0.2	Dimethylaniline monooxygenase. InterPro: Flavin monooxygenase FMO	
*AAEL004278*	11/12	2.7 ± 1.2	GO: Lipid metabolic process, Integral component of membrane, Oxidoreductase activity, Heme binding	
*AAEL000024*	10/12	1.9 ± 0.8	Dopachrome-conversion enzyme, putative	
*AAEL014246*	9/12	1.5 ± 0.3	Glucosyl/glucuronosyl transferases	
*AAEL003832*	11/12	11.7 ± 6.7	Defensin anti-microbial peptide (*DEFC*)	Innate immunity
*AAEL015458*	11/12	14.4 ± 12.1	Transferrin (*Tf1*)	
*AAEL011453*	10/12	2.8 ± 1.1	C-Type Lectin (*CTL14*)	
*AAEL011621*	10/12	2.8 ± 1.1	C-Type Lectin - mannose binding (*CTLMA13*)	
*AAEL009474*	10/12	3.1 ± 1.5	Peptidoglycan Recognition Protein (Short) (*PGRPS1*)	
*AAEL006516*	10/12	1.3 ± 0.2	Vacuolar ATP synthase subunit h	Trans- membrane transport
*AAEL010819*	9/12	1.3 ± 0.2	Vacuolar ATP synthase subunit H	
*AAEL000291*	9/12	1.2 ± 0.1	V-type proton ATPase 16 kDa proteolipid subunit	
*AAEL010139*	11/12	16.9 ± 28.8	Serine protease, putative	Proteolysis
*AAEL013032*	10/12	2.2 ± 1.3	GO: Serine-type endopeptidase activity, Proteolysis. InterPro: Trypsin_dom	
*AAEL003963*	9/12	1.4 ± 0.2	Calpain 4, 6, 7, invertebrate	
*AAEL001082*	10/12	1.8 ± 0.6	InterPro: Myofilin (IPR031828)	Other
*AAEL006507*	9/12	1.5 ± 0.3	GO: Methyltransferase activity. InterPro: RNA methyltransferase Bin3-like	
*AAEL008471*	9/12	1.4 ± 0.2	GO: Protein targeting, Intracellular protein transport, ATP binding. InterPro: SecA	
*AAEL003417*	9/12	1.4 ± 0.2	Mitochondrial ATPase inhibitor, putative	

a Mean Fold-changes ± Standard Deviation. The reference condition for Fold-change calculation is *Wolbachia*-uninfected.

**Table 3:** Genes consistently downregulated by *Wolbachia* in *A. aegypti*.

Gene ID	# significant tests (*p*<0.05)	Mean Fold-change^a^	Description	Category
*AAEL014016*	9/12	1.2 ± 0.1	Breast cancer metastasis-suppressor. InterPro: Sds3-like	Nucleic acid interaction
*AAEL012392*	8/12	1.3 ± 0.2	GO: Nucleus, Nucleic acid binding, Zinc ion binding	
*AAEL010797*	8/12	1.2 ± 0.1	Mediator of RNA polymerase II transcription subunit 21 (Med21)	
*AAEL003490*	8/12	1.3 ± 0.2	Zinc finger-tcix domain-containing protein	
*AAEL009650*	8/12	1.3 ± 0.2	GO: Nucleic acid binding, 3’-5’ exonuclease activity	
*AAEL012989*	8/12	1.2 ± 0.1	Nuclear pore complex protein Nup85	Other
*AAEL014347*	8/12	1.3 ± 0.2	GO: Integral component of membrane, Calcium ion binding, Protein binding	
*AAEL004308*	8/12	1.1 ± 0.1	Proteasome subunit alpha type	
*AAEL014816*	8/12	1.3 ± 0.2	Uncharacterized product	
*AAEL012148*	8/12	1.6 ± 0.6	Uncharacterized product	

a Mean Fold-changes ± Standard Deviation. The reference condition for Fold-change calculation is *Wolbachia*-infected.

**Table 4:** GO terms consistently up- and downregulated by *Wolbachia* in *A. aegypti*.

Category	GO terms	Expression pattern
Electron transport chain	Respirasome, Respiratory chain complex, Oxidoreduction-driven active transmembrane transporter activity, Oxidative phosphorylation, ATP synthesis coupled electron transport, Respiratory electron transport chain, Electron transfer activity, Mitochondrial inner membrane, Mitochondrial respirasome, Aerobic electron transport chain, Mitochondrial ATP synthesis coupled electron transport, Oxidoreductase complex.	Upregulated in all infected groups except for *w*MelPop-infected heads and muscles from Ye et al. (2013).
Carbohydrate transmembrane transport	Carbohydrate transmembrane transporter activity, Sugar transmembrane transporter activity, Carbohydrate transmembrane transport.	Upregulated in all infected groups except for *w*MelPop-infected heads and muscles from Ye et al. (2013).
Serine-type peptidase activity	Serine hydrolase activity, Serine-type peptidase activity, Serine-type endopeptidase activity	Upregulated in all infected groups except for *w*Mel-infected midguts from Boehm et al. (2023).
Serine-type peptidase inhibition	Serine-type endopeptidase inhibitor activity, Negative regulation of catalytic activity, Negative regulation of molecular function, Negative regulation of protein metabolic process	Upregulated in all infected groups except for *w*Mel-infected midguts from Boehm et al. (2023).
DNA replication	DNA replication	Downregulated in all infected groups except for *w*MelPop-infected heads and muscles from Ye et al. (2013).

We found 21 consistently upregulated genes related to metabolism, innate immunity, transmembrane transport, proteolysis and other functions (**Table 2**). Metabolism-related genes included two putative farnesoic acid O-methyltransferases, involved in the synthesis of juvenile hormones (Yagi et al., 1991), a flavin-dependent monooxygenase, involved in detoxification (Bailleul et al., 2023) and a putative dopachrome conversion enzyme, involved in melanin biosynthesis (Jones et al., 2014). Innate immunity-related genes comprised two C-type lectins and a PGRP, which are pathogen pattern recognition genes, as well as the extracellular iron transporter transferrin which serves immune functions by limiting pathogen access to iron (Iatsenko et al., 2020), and the antimicrobial peptide defensin. Transmembrane transport genes comprised three subunits of vacuolar ATP synthase, which is a proton pump responsible for the acidification of intracellular organelles (Huang et al., 2006). Additional genes included a putative mitochondrial ATPase inhibitor, a putative RNA-methyltransferase and myofilin, which is a component of the thick filaments of insect striated muscles (Qiu et al., 2005).

We also found 10 consistently downregulated genes (**Table 2**) mainly related to nucleic acid interaction, including a Sds3-like gene [which are implicated in repression of gene expression (Jones et al., 2014)], Med21 (a component of the Mediator of transcriptional regulation, which mediates the interaction of RNA polymerase II and transcription activators or repressors (Baumli et al., 2005)), and a putative RNA-exonuclease. Additional genes included a nuclear pore protein, a proteasome subunit and two genes with uncharacterized function.

Consistently upregulated GO terms were associated to the electron transport chain, carbohydrate transmembrane transport, serine-type peptidase activity and serine-type peptidase inhibition, while the GO term DNA replication was consistently downregulated (**Table 3**).

## 4 Discussion

*Wolbachia*-based arboviral disease control strategies have been successfully implemented in Indonesia, Australia and Brazil, however mechanistic understanding of the *A. aegypti-Wolbachia* system is still lacking. This work provides new insights on previously published transcriptomic datasets and identifies host genes and functions commonly affected by *Wolbachia* transfection, transcending differences such as tissue, age and bacterial strain, thus providing a valuable resource for guiding future experimental works towards the most promising avenues. Main findings and hypotheses, and their relevance for arboviral control, are summarized in **Table 5** and detailed in the following sections of this discussion.

**Table 5:** Main findings of this work and their relevance for arboviral control.

Novel observations	Relevant background	Novel hypotheses	Relevance of these findings and hypotheses for arboviral control
Downregulation of six opsins by *w*MelPop in *A. aegypti* heads.	Opsins mediate mosquito vision and light driven behavior, including feed seeking (Zhan et al., 2021; Liu et al., 2022). *w*MelPop attenuates photofobicity of *A. aegypti* larvae (Suh and Dobson, 2013).	Downregulation of opsins causes loss of photofobicity in *w*MelPop *A. aegypti* larvae. *w*MelPop impacts *A. aegypti* feed seeking ability.	Larval photofobicity and adult feed seeking ability impact mosquito fitness, which in turn determines the success of *Wolbachia* introgression for arboviral control.
*Wolbachia* consistently upregulates the host electron transport chain (ETC). Dual- and NADPH oxidases are not upregulated across datasets.	ETC activity is a main source of reactive oxygen species (ROS) in cells (Nolfi-Donegan et al., 2020). ROS production is considered a key mechanism for pathogen blocking (Brennan et al., 2008; Pan et al., 2012; Wong et al., 2015; Liu et al., 2016). Dual- and NADPH oxidases were previously proposed as main host factors for *Wolbachia*-induced ROS production (Pan et al., 2012).	ETC upregulation, instead of dual- and NADPH oxidases, is the main host factor for *Wolbachia*-induced ROS production.	As ROS production is considered a key mechanism for pathogen blocking, understanding its underlying mechanisms will help to preserve the phenotype.
*Wolbachia* consistently upregulates two host vacuolar ATPase (v-ATPase) subunits	v-ATPases are proton pumps that acidify intracellular membranous organelles, such as vacuoles (Huang et al., 2006). *Wolbachia* lives inside host vacuoles (Cho et al., 2011; Fattouh et al., 2019). v-ATPases have proviral effects in *A. aegypti*, promoting viral RNA release into the cytoplasm (Sessions et al., 2009; Kang et al., 2014). *Wolbachia* has shown pathogen enhancement in *Culex* mosquitoes (Dodson et al., 2014).	v-ATPase upregulation is a proviral effect induced by *Wolbachia*, masked by other antiviral effects.	Proviral effects by *Wolbachia* need careful consideration, as they may constitute the basis for pathogen enhancement. Avoiding this phenotype will benefit from its mechanistic understanding.
*Wolbachia* consistently upregulates two putative farnesoic acid methyl transferases (FAMeTs) in *A. aegypti.*	FAMeTs catalyze a late step in juvenile hormone (JH) biosynthesis (Zou et al., 2013; Nouzova et al., 2021). JHs impact development, reproduction and gene expression in *A. aegypti* (Zou et al., 2013; Liu et al., 2014; Noriega, 2014) and their alterations may contribute to CI in *Drosophila* (Liu et al., 2014). Methyl farnesoate, the direct product of FAMeTs, can partially substitute the functions of JHs (Nouzova et al., 2021).	FAMeT upregulation by *Wolbachia* has deep effects on *A. aegypti* physiology, mediated by JH or methyl farnesoate activity. FAMeT upregulation by *Wolbachia* also occurs in male mosquitoes and impacts CI.	Mosquito physiology impacts fitness, which in turn determines the success of *Wolbachia* introgression for arboviral control. CI is a key phenotype for *Wolbachia*’s introgression. Preserving CI will benefit from its mechanistic understanding.
*Wolbachia* consistently upregulates a flavin-containing monooxygenase (FMO) in *A. aegypti*.	FMOs oxygenate nucleophilic nitrogen, sulphur, phosphorus and selenium atoms, and are implicated in the neutralization of insecticides (Sehlmeyer et al., 2010; Tian et al., 2018; Mallott et al., 2019). Coordinating *Wolbachia*- and insecticide-based interventions is key for the success of arboviral control schemes (Hoffmann and Turelli, 2013; Garcia et al., 2020).	*Wolbachia* provides *A. aegypti* with resistance to certain insecticides.	If FMOs upregulation confers resistance to certain insecticides, their use might be considered to enhance *Wolbachia* introgression, avoiding introduction of new insecticide-resistant alleles.
*Wolbachia* consistently downregulates genes with nucleic acid-related functions in *A. aegypti*. *Wolbachia* consistently downregulates putative exonuclease *AAEL009650*.	RNA viruses heavily depend on host RNA-interacting genes for the success of their life cycle (Sessions et al., 2009; Viktorovskaya et al., 2016; Wichit et al., 2021). *AAEL009650* is homologous to *Drosophila* exonuclease-like *CG6744*, which contributes to DENV infection (Sessions et al., 2009).	Downregulation of RNA-interacting genes mediates pathogen blocking.	Pathogen blocking is a key phenotype for the success of *Wolbachia*-based arboviral control. Understanding its genesis will help to preserve this phenotype.
*Wolbachia* consistently upregulates defensin *DEFC*, transferrin *Tf1*, peptidoglycan recognition protein *PGRPS1*, serine-type peptidases and their inhibitors. Canonical genes from the Toll, IMD and JAK/STAT pathways are inconsistently regulated or primarily downregulated.	Immune priming is considered a key mechanism of pathogen blocking (Kambris et al., 2009; Moreira et al., 2009; Pan et al., 2012; Rancès et al., 2012; Terradas et al., 2017). Particularly, *DEFC* and *Tf1* have proven antiviral effects in *A. aegypti* (Pan et al., 2012; Zhu et al., 2019).	Overexpression of *DEFC* and *Tf1* results from conflicting transcriptomic mechanisms, with silencing promoted by downregulation of *TUBE*, *Rel1A* and *IKK1*, and induction promoted by upregulation of *PGRPS1* and serine-protease cascades, further supported by non-transcriptomic effects.	As immune priming is key for pathogen blocking, understanding its underlying mechanisms will help to preserve this phenotype.

### 4.1 New functional insights on the effects of *Wolbachia* in *A. aegypti*

*De novo* analyses performed in this work revealed functional impacts of *Wolbachia* transfection in *A. aegypti* that remained concealed within published datasets. These included upregulation of transport processes in data from Kambris et al. (2009), downregulation of cell cycle and DNA metabolism in Kambris et al. (2009) and Rancès et al. (2012), and upregulation of respirasome in Boehm et al. (2023). The analysis of the dataset from Ye et al. (2013) is of particular interest as it comprises the only genome-wide transcriptomic data of *w*MelPop-infected *A. aegypti* heads published so far, posing it as a unique source of tissue-specific mechanistic insight. We found that *w*MelPop downregulated six long and short wavelength opsins, light sensitive receptors that initiate phototransduction and mediate visual perception in mosquitoes from larval to adult stages (Rocha et al., 2015; Giraldo-Calderón et al., 2017). A coordinated downregulation of opsins has been previously identified for the native *Wolbachia* infection of *Aedes fluviatilis* (Caragata et al., 2017), but not for *A. aegypti* transinfection.

It has been shown that *w*MelPop attenuates photofobicity (*i.e.*, light avoidance) in *A. aegypti* larvae (Suh and Dobson, 2013) and that inhibition of opsins impairs visual recognition of feeding targets in adult *A. aegypti* (Zhan et al., 2021). Considering these reports in the light of our findings, we propose downregulation of opsins as a feasible mechanism for photofobicity attenuation by *w*MelPop and predict that this strain impairs *A. aegypti*’s feeding target seeking. As photophobicity and feed seeking ability are relevant for mosquito fitness at multiple developmental stages —the first one reducing predation and desiccation risk for larvae (Liu et al., 2022) and the second one allowing adult females to obtain blood meals (Zhan et al., 2021)— testing these hypotheses will help clarify mechanistic bases of the dramatic fitness costs induced by *w*MelPop, which hinder its use for population replacement (Allman et al., 2020).

### 4.2 *Wolbachia* consistently upregulates electron transport chain genes in *A. aegypti*

Several GO terms associated with host electron transport chain (ETC) and carbohydrate transmembrane transport were found to be upregulated by *Wolbachia* in 10 out of the 12 analyzed datasets. ETC is a central step in mitochondrial aerobic respiration, responsible for most of adenosine triphosphate (ATP) production in the cell and is fueled by glycolysis. Upregulation of host ETC and carbohydrate transmembrane transport can be interpreted as a tendency to increase ATP production along with the availability of sugars to fuel this process. Consistent upregulation of the putative mitochondrial ATPase inhibitor *AAEL003417* also identified in this work may be a compensatory mechanism to control ATP production.

While it has been suggested that *Wolbachia* provides hosts with ATP from its own bacterial aerobic respiration (Darby et al., 2012; Gill et al., 2014), to the best of our knowledge our study is the first to show the persistence of host ETC upregulation by *Wolbachia* in *A. aegypti*. Interestingly, *Wolbachia* has a complete biosynthetic pathway for heme synthesis and may provide it to its hosts (Gill et al., 2014). As ETC heavily depends on heme, it could be hypothesized that *Wolbachia* supports host ETC upregulation by providing this cofactor.

Upregulation of ETC can also be linked to *Wolbachia*’s induction of reactive oxygen species (ROS), which has been reported in *Aedes* hosts (Brennan et al., 2008; Pan et al., 2012; Caragata et al., 2016) and is considered a key phenomena for antiviral defense in *Diptera* (Brennan et al., 2008; Pan et al., 2012; Wong et al., 2015; Liu et al., 2016). Electron leakage, which is an incomplete reduction of oxygen to form superoxide (O_2_^-^) due to ETC activity, is a major source of cellular ROS (Nolfi-Donegan et al., 2020). We propose that a generalized upregulation of ETC genes is a potential persistent host mechanism behind ROS induction by *Wolbachia* in *A. aegypti*. Additionally, as the extent of electron leakage is modulated by mitochondrial ATPase activity (Beck et al., 2016; Nolfi-Donegan et al., 2020), the consistent upregulation of putative mitochondrial ATPase inhibitor *AAEL003417* found here might be further modulating ROS production.

It has been previously proposed that the main *A. aegypti* factors responsible for *Wolbachia-*induced ROS are dual oxidases and NADPH-oxidases (Pan et al., 2012; Zug and Hammerstein, 2015), which drive an immune oxidative burst in *Drosophila* under microbial infection (Ha et al., 2005). Surprisingly, we found that NADPH-oxidase *AAEL002039* was insignificant (*p*>0.05) in 11 of 12 tests, while all six significant results for dual oxidase *AAEL007563* and its maturation factor *AAEL007562* corresponded to downregulation (**Supplementary Table 4**). Typical downregulation of *AAEL007562* and *AAEL007563* could constitute a host mechanism to counterbalance oxidative stress associated with other mechanisms such as the bacterial aerobic respiration and, as previously discussed, host ETC upregulation.

Our results contrast with the 28-fold increase in *AAEL007562* transcripts reported by Pan et al. (2012) for *w*AlbB-infected carcasses of non-blood fed *A. aegypti* (Pan et al., 2012), a discordance that may be attributed to the difference of *Wolbachia* strains or specific biological conditions. On the other hand, our results are consistent with the study of Caragata et al. (2016) on *w*Mel-infected *A. aegypti* whole bodies, where ROS induction was verified without upregulation of *AAEL007562* (Caragata et al., 2016) and further supports an alternative mechanism for typical ROS production such as enhanced host ETC activity. This does not exclude the possibility that dual- and NADPH-oxidases plays a role in pathogen blocking by producing ROS early after a blood meal, which may have been missed in the transcriptomic datasets from Boehm et al. (2023) analyzed in this work, as they start at 4 days post blood feeding.

Antiviral ROS induction may be dynamically driven by multiple host mechanisms, including dual- and NADPH-oxidase upregulation and host ETC upregulation, as well as bacterial mechanisms such as its own energy metabolism. In order to distinguish the contributions of such mechanisms, new molecular screenings are needed that: (i) provide extended time coverage and resolution surrounding viral ingestion, (ii) couple expression data to oxidative stress markers, and (iii) selectively affect components of the different mechanisms (e.g. via interference RNA).

### 4.3 *Wolbachia* consistently upregulates vacuolar ATP synthase subunits in *A. aegypti*

We found three consistently upregulated vacuolar ATP synthase (v-ATPase) subunits: *AAEL006516*, *AAEL010819* and *AAEL000291*. v-ATPases are proton pumps that acidify intracellular membranous organelles, such as lysosomes, endosomes and parasitophorous vacuoles (Huang et al., 2006). As *Wolbachia* lives inside host-derived vacuoles (Cho et al., 2011; Fattouh et al., 2019), consistent upregulation of v-ATPases may have direct impacts in the immediate environment of the bacterium. v-ATPases are also responsible for the acidification of DENV-containing endosomes, which is a requisite for the release of viral RNA into the cytoplasm for further translation and replication. Consequently, inhibition of v-ATPases has proven antagonic to DENV infection in *Aedes* mosquitoes, *Aedes* cells and *HeLa* cells (Krishnan et al., 2007; Sessions et al., 2009; Kang et al., 2014). Therefore, v-ATPase upregulation may be a pro-viral effect of *Wolbachia*, obscured however by its repertoire of antiviral effects. Existence of pro-viral *Wolbachia* effects should be carefully considered, as overall enhancement of arbovirus infection by *Wolbachia* is possible (Dodson et al., 2014). Specifically, disruption of v-ATPase activity by RNAi or v-ATPase inhibitors hold potential to clarify the consequences of *Wolbachia*’s upregulation of host v-ATPases for viral infection in *A. aegypti*.

### 4.4 *Wolbachia* consistently upregulates two putative farnesoic acid methyl transferases in *A. aegypti*

We found the consistent upregulation of two putative farnesoic acid methyl transferases (FAMeTs), *AAEL001508* and *AAEL004667*, which catalyze a late step in the biosynthesis of insect juvenile hormones (Zou et al., 2013; Nouzova et al., 2021). Interactions of *Wolbachia* with the juvenile hormone biosynthetic pathway have been previously reported in *Diptera* (Liu et al., 2014; Ramos et al., 2022), but to the best of our knowledge, this is the first study showing *Wolbachia*’s consistent upregulation of putative *A. aegypti*’s FAMeTs. Juvenile hormone III (JHIII) is a key regulator of development and reproduction in *A. aegypti*, and its titer determines the expression of thousands of genes (Zou et al., 2013; Liu et al., 2014; Noriega, 2014). Also, the direct product of FAMeT —methyl farnesoate— can partially substitute the functions of JHIII in *A. aegypti* (Nouzova et al., 2021) and possibly serve additional uncharacterized functions (Van Ekert et al., 2014). In light of these considerations, either by altering juvenile hormone or methyl farnesoate production, upregulation of FAMeTs could have deep consequences for *A. aegypti* physiology. Further experiments are needed to confirm the enzymatic activity of *AAEL001508* and *AAEL004667*, as well as to unveil their specific roles in the *A. aegypti*-*Wolbachia* symbiosis at different life stages. Checking if this transcriptomic phenomena observed for female mosquitoes has a parallel in males would be of particular interest, as alterations of JHIII have been proposed as a contributory mechanism for *Wolbachia*-induced CI in *Drosophila* (Liu et al., 2014).

### 4.5 *Wolbachia* consistently upregulates a flavin-containing monooxygenase implicated in xenobiotic metabolism in *A. aegypti*

We found the consistent upregulation of flavin-containing monooxygenase (FMO) *AAEL000829*, which catalyzes the incorporation of an oxygen atom from O_2_ to another molecule, using flavin adenine dinucleotide as a prosthetic group (Sehlmeyer et al., 2010; Torres Pazmiño et al., 2010). Another FMO, *AAEL000834*, shows a similar profile of expression being upregulated in 8 of the 9 significant tests (*p*<0.05) (**Supplementary Table 4**). FMOs catalyze the oxygenation of nucleophilic nitrogen, sulphur, phosphorus and selenium atoms (Jones et al., 2014), and are implicated in the neutralization of xenobiotics (i.e. foreign substances) (Bailleul et al., 2023), particularly insecticides (Sehlmeyer et al., 2010; Tian et al., 2018; Mallott et al., 2019). Although direct effects of *Wolbachia* on *A. aegypti*’s resistance to typical insecticides is not clear (Endersby and Hoffmann, 2013), the consistent upregulation of FMOs found here opens the question if there are additional substances to which *Wolbachia*-infected *A. aegypti* is more resistant than its uninfected counterpart.

Coordinating *Wolbachia*- and insecticide-based vector control methods is important for the overall success of arboviral control (Hoffmann and Turelli, 2013; Garcia et al., 2020). Particularly, coupling *Wolbachia*-introduction with that of insecticide resistance genotypes has been proposed as a way to facilitate spread of this bacteria in native mosquito populations, a paramount task for the use of strains that cause high fitness costs such as *w*MelPop (Hoffmann and Turelli, 2013; Garcia et al., 2020). If the consistent upregulation of FMOs confers resistance to specific compounds in *Wolbachia*-infected *A. aegypti*, their controlled use might be considered to enhance *Wolbachia* introgression in native *A. aegypti* populations. By leveraging an effect already present in *Wolbachia*-infected *A. aegypti*, this could avoid the introduction of new insecticide-resistant alleles in native populations, a major concern regarding the use of insecticides for the enhancement of *Wolbachia*-based arboviral control (Hoffmann and Turelli, 2013; Garcia et al., 2020).

### 4.6 *Wolbachia* consistently downregulates genes with nucleic acid-related functions in *A. aegypti*

Our results suggest a coordinated host metabolic shift towards enhanced aerobic energy metabolism and reduced cellular proliferation, as DNA replication GO term was downregulated by *Wolbachia* in 10 out of 12 tests and, as previously discussed, a consistent upregulation of ETC and carbohydrate transmembrane transport was found. This scenario may be induced by *Wolbachia* to favor its own preservation in *A. aegypti* adult stages such as those considered in this study, however, it is foreseeable that a similar effect in earlier developmental stages would critically impact development. It would be therefore interesting to study if this downregulation also stands in earlier stages of *A. aegypti* development, pinpointing the need for genome-wide transcriptomic analyses of *Wolbachia*-infected *A. aegypti* at new biological conditions.

RNA viruses such as DENV, ZIKV and CHIKV have small genomes and heavily depend on host factors for the success of their life cycle, including various host RNA-interacting genes (Sessions et al., 2009; Viktorovskaya et al., 2016; Wichit et al., 2021). Our results suggest that downregulation of genes with nucleic acid interaction is not restricted to DNA but also includes RNA-interacting genes. For instance, the mRNA metabolic process GO term had a negative Normalized Enrichment Score in 10 out of 12 tests, 7 of which were significant (*p*<0.05) (**Supplementary Table 4**).

Consistent downregulation of DNA- and RNA-interacting genes by *Wolbachia* has not been discussed in individual studies (Kambris et al., 2009; Rancès et al., 2012; Ye et al., 2013; Boehm et al., 2023; Wimalasiri-Yapa et al., 2023), likely due to the small magnitude of their fold-changes. Our meta-analysis offers the statistical power required for capturing such subtle tendencies, opening the field for new mechanistic hypotheses. Particularly, downregulation of RNA-interacting genes by *Wolbachia* may constitute an unexplored mechanism for pathogen blocking, explaining the broad-range of RNA viruses to which *Wolbachia* offers protection. Notably, the consistently downregulated gene *AAEL009650* is homologous to *Drosophila* exonuclease-like *CG6744*, which contributes to DENV infection (Sessions et al., 2009). This poses *AAEL009650* as a concrete molecular target for investigating a potential link between downregulation of nucleic acid-interacting genes and pathogen blocking.

### 4.7 A consistent signature of immune priming by *Wolbachia* in *A. aegypti*

Mosquitoes lack adaptive immunity and thus rely on innate immunity components for fighting arboviruses, such as downstream effectors of the Toll, IMD, JAK/STAT and RNAi signaling pathways (Xi et al., 2008b; Sim et al., 2013; Angleró-Rodríguez et al., 2017; Chowdhury et al., 2020). Evidence of innate immunity pre-activation (*priming*) by *Wolbachia* includes upregulation of signaling components from the mentioned pathways (*e.g. Rel1* and *Rel2* for the Toll and IMD pathways, respectively) and several of their downstream elements (*e.g.* immune effectors defensin, cecropin and transferrin (Kambris et al., 2009; Pan et al., 2012; Rancès et al., 2012; Caragata et al., 2019) and antioxidant proteins (Pan et al., 2012)). As immune priming is considered a key mechanism of pathogen blocking (Kambris et al., 2009; Moreira et al., 2009; Pan et al., 2012; Rancès et al., 2012; Terradas et al., 2017), understanding its transcriptomic basis is essential. Our analysis contributes to this characterization, identifying immunity-related genes that are consistently affected across datasets and highlighting that many canonical components of immune pathways are inconsistently affected or primarily downregulated.

We found consistent upregulation of defensin *DEFC*, transferrin *Tf1*, peptidoglycan recognition protein *PGRPS1* and GO terms related to serine-type peptidase activity and inhibition. This is in agreement with *Wolbachia*’s priming of the Immunodeficiency (IMD) and Toll pathways in *A. aegypti* (Rancès et al., 2012), as expression of transferrin (an iron binding protein which reduces iron availability for pathogens (Iatsenko et al., 2020)) and the antimicrobial peptide defensin is inducible by them (Costa et al., 2009; Iatsenko et al., 2020). Also, these pathways can be activated extracellularly by the action of pattern recognition receptors such as *PGRPS1*, which elicit protease signaling cascades mediated by serine-type peptidases and serine-type peptidase inhibitors (Kanost and Jiang, 2015). The expression pattern of *Rel2* observed here is also consistent with the activation of the IMD pathway, as it has a positive expression change in 10 out of 12 tests, 9 of which are significant (*p*<0.05) (**Supplementary Table 5**).

However, we note that most canonical genes from the Toll and IMD pathways (which comprise 23 and 8 known *A. aegypti* genes, respectively (Kanehisa et al., 2023)) are inconsistently affected across datasets, and some genes show a tendency towards downregulation. Particularly, for the Toll pathway, *TUBE* has a negative expression change in 9 out of 12 tests, 6 of which are significant (*p*<0.05), while *Rel1A* has a negative expression change in 7 out of 12 tests, 6 of which are significant (*p*<0.05) (**Supplementary Table 5**). *IKK1*, part of the IMD pathway, has a negative expression change in 10 out of 12 tests, 3 of which are significant (*p*<0.05) (**Supplementary Table 5**). Overall, our results show that *Wolbachia* consistently induces downstream effectors of the Toll and IMD pathways (*DEFC* and *Tf1*), but this is not readily explained by upregulation of canonical signaling genes as, with the exception of *Rel2*, these are inconsistently affected or primarily downregulated. Expression of these effectors may result from conflicting transcriptomic mechanisms, where silencing is promoted by downregulation of *TUBE*, *Rel1A* and *IKK1*, and induction is promoted by upregulation of *PGRPS1* and serine-protease cascades, further supported by non-transcriptomic effects such as nuclear translocation of the transcription factor NF-kB due to oxidative stress [this last mechanism has been previously proposed in (Pan et al., 2012)]. Janus Kinase/Signal Transducer Activators of Transcription (JAK/STAT) pathway’s role in *Wolbachia*-mediated pathogen blocking has also been hypothesized (Pan et al., 2018), as components of this pathway were upregulated by *Wolbachia* in *Drosophila* (Xi et al., 2008a), and this pathway restricts DENV in *A. aegypti* (Souza-Neto et al., 2009) and West Nile Virus in Culex mosquitoes (Paradkar et al., 2012). We found that JAK/STAT activators (*DOME*, *HOP*, *STAT*) and inhibitors (*SOCS*, *PIAS1*) are mainly downregulated, as from the 26 significant tests that they accumulate (*p*<0.05), 23 correspond to downregulation (**Supplementary Table 5**). Again, this suggests that there are conflicting transcriptomic mechanisms regarding immune priming of the JAK/STAT pathway by *Wolbachia*.

Knowing the specific mechanisms underlying *Wolbachia*’s immune priming is crucial for understanding pathogen blocking by *Wolbachia* in *A. aegypti*, as it drives the expression of key antiviral factors such as *DEFC* (Pan et al., 2012) and transferrins (Zhu et al., 2019). Our work contributes in identifying consistent transcriptional regulations associated with immune priming by *Wolbachia* in *A. aegypti*. We also highlight that there are transcriptomic mechanisms opposing immune activation. These require careful consideration and monitoring, as they may constitute a basis for attenuation of pathogen blocking in the future, a main concern regarding *Wolbachia*-based arboviral control.

### 4.8 Limitations of this work

Given the early stage of mechanistic research into the *A. aegypti-Wolbachia* symbiosis and the wide variety of reported *Wolbachia* effects in this mosquito, identifying the most promising avenues for further investigation is crucial for an effective allocation of experimental resources. Although wet-lab confirmation would further reinforce our conclusions, this meta-analysis offers substantial value even in its absence. Consistent effects are particularly relevant targets, as they potentially represent fundamental interactions that would be active under any *Wolbachia*-based arboviral control implementation scenario. Our meta-analysis is the first to identify such consistently affected genes and functions, as well as providing links with relevant phenotypes for arboviral control, including pathogen blocking. Furthermore, to compensate for lack of experimental validation, a conservative approach (maxP) (Chang et al., 2013; Li and Ghosh, 2014; Yoon et al., 2021) was selected for *p*-value combination, thus strengthening our confidence in the identified genes and pathways.

While heterogeneity of datasets widens the scope of our analysis, it also limits the kind of conclusions that can be derived from it. Several biological conditions are addressed by a single study [*e.g.* mosquito heads and muscles in Ye et al. (2013) (Ye et al., 2013), carcasses and midguts in Boehm et al. (2023) (Boehm et al., 2023), and native *A. aegypti* populations with *Wolbachia* introgression in Wimalasiri-Yapa et al. (2023) (Wimalasiri-Yapa et al., 2023)], hindering inter-study comparisons for assessing the effects of factors different from *Wolbachia*’s presence. Such disentanglement of effects would be of great value for a fine-grained understanding of *Wolbachia*’s behavior under specific circumstances, which motivates obtention of data from the remaining factor level combinations.

Acknowledging the limitations of currently available data, we aimed at answering a more coarse-grained but equally valid question: which host genes and functions have the greatest evidence of being always affected by *Wolbachia* across the considered studies? A *p*-value meta-analysis provides the flexibility to answer such questions exclusively based on *p*-values.

All collected transcriptomic data corresponded to adult female mosquitoes, a bias that was not introduced by our exclusion criteria but reflects a research focus on these individuals, which are directly responsible for arboviral transmission through biting (Zahid et al., 2023). Considering this as well as the relevance of transcriptomics for understanding mechanisms behind symbiosis, we highlight the need for expanding these screenings to additional biological conditions that are crucial for *Wolbachia*-based arboviral control. For example, *Wolbachia*-induced fitness disadvantages are mainly manifested at egg and larval stages (Suh and Dobson, 2013; Farnesi et al., 2019; Allman et al., 2020) and, as transfected males are the agents of cytoplasmic incompatibility, their fitness is crucial for the prevalence of *Wolbachia* in mosquito populations (Turley et al., 2013). Although the bias on current datasets limits the scope of our findings to a specific biological condition, we were able to relate them to additional conditions via hypotheses based on previous literature, as we did for downregulation of opsins (relating it to loss of photofobicity in infected *A. aegypti* larvae) and FAMeTs upregulation (relating it to CI in infected *A. aegypti* males). Addressing the relevance of additional symbiotic conditions requires new experimental efforts, which can be guided by the findings and hypotheses provided by this work.

## 5 Conclusion

Genome-wide omic datasets from related conditions accumulate in time and bioinformatic resources enabling their interpretation evolve. This offers the opportunity to extract new value from previously published datasets by both analyzing them with updated resources, and comparing older to newer datasets by meta-analysis methods, offering global insights that no study by its own can provide.

In this work, by performing a *de novo* meta-analysis of transcriptomic data we were able to identify novel effects and a transcriptomic signature of *Wolbachia* in *A. aegypti*, which can explain previously observed phenotypes and provide new hypotheses and relevant targets for further study. We highlight the relevance of raw data availability, as identification of common effects at functional level was mediated by gene set enrichment analyses that were not performed originally, and that would not have been obtained from pre-processed data such as filtered lists of differentially expressed genes. Follow-up work on these observations holds promise in producing valuable information towards preventing pathogen enhancement and loss of pathogen blocking, coupling *Wolbachia*-based control with other strategies, and enabling the use of specific *Wolbachia* strains, thus contributing towards translational research for sustainability of *Wolbachia*-based arboviral control.

## Data Availability

Publicly available datasets were analyzed in this study. This data can be found here: https://www.ncbi.nlm.nih.gov/geo/query/acc.cgi?acc=GSE17469 (Gene Expression Omnibus: Accession GSE17469). https://www.ebi.ac.uk/biostudies/arrayexpress/studies/E-MEXP-2931?query=E-MEXP-2931 (ArrayExpress: Accession E-MEXP-2931). https://www.ebi.ac.uk/biostudies/arrayexpress/studies/E-MEXP-2907?query=E-MEXP-2907 (ArrayExpress: Accession E-MEXP-2907). https://www.ncbi.nlm.nih.gov/sra/?term=PRJNA867516 (Sequence Read Archive: Accession PRJNA867516). https://www.ncbi.nlm.nih.gov/sra/?term=PRJNA949154 (Sequence Read Archive: Accession PRJNA949154).

## Supplementary material

Supplementary Table 1 Datasets included and excluded from this study.

Supplementary Table 2 RNA-Seq mapping statistics. Number of reads in each RNA-Seq dataset and percentage of reads mapped to the *A. aegypti* reference genome.

Supplementary Table 3 Gene set enrichment analysis results. Significant GSEA results for each gene list derived from an infected versus uninfected comparison. GSEA leading edges are filtered to include only differentially expressed genes (*p*<0.05).

Supplementary Table 4 Meta-analysis results. Significant results from meta-analysis of *p*-values derived from GSEA and differential expression analyses.

Supplementary Table 5 Expression pattern of genes from the Toll, IMD and JAK/STAT pathways. Log2 expression fold-changes (*Wolbachia*-infected vs uninfected) for *A. aegypti* genes in the Toll, IMD and JAK/STAT pathways according to the Kyoto Encyclopedia of Genes and Genomes. nt: not tested, **p*<0.05.

Supplementary Figure 1 Microarray MA-plots. MA-plots of two-channel microarray data from **(A)** Kambris et al. (2009), **(B)** Rancès et al. (2012) and **(C)** Ye et al. (2013). MA-plots show M values (logarithmic fold-change between red and green intensities for each spot) versus A values (average of logarithmic red and green intensities for each spot). Control spots are colored (see Legend).

Supplementary Figure 2 Principal component analysis and hierarchical biclustering of transcriptomic datasets. *De novo* analysis of datasets from **(A)** Kambris et al. (2009), **(B)** Rancès et al. (2012), **(C)** Ye et al. (2013), **(D)** Wimalasiri-Yapa et al. (2023) and **(E)** Boehm et al. (2023).
