# Supplementary figures and images for: Unveiling Wolbachia transcriptomic signature in the arboviral vector Aedes aegypti
Source: Front Cell Infect Microbiol. 2025 Apr 28;15:1538459. doi: 10.3389/fcimb.2025.1538459 (PMC12066770; doi:10.3389/fcimb.2025.1538459)

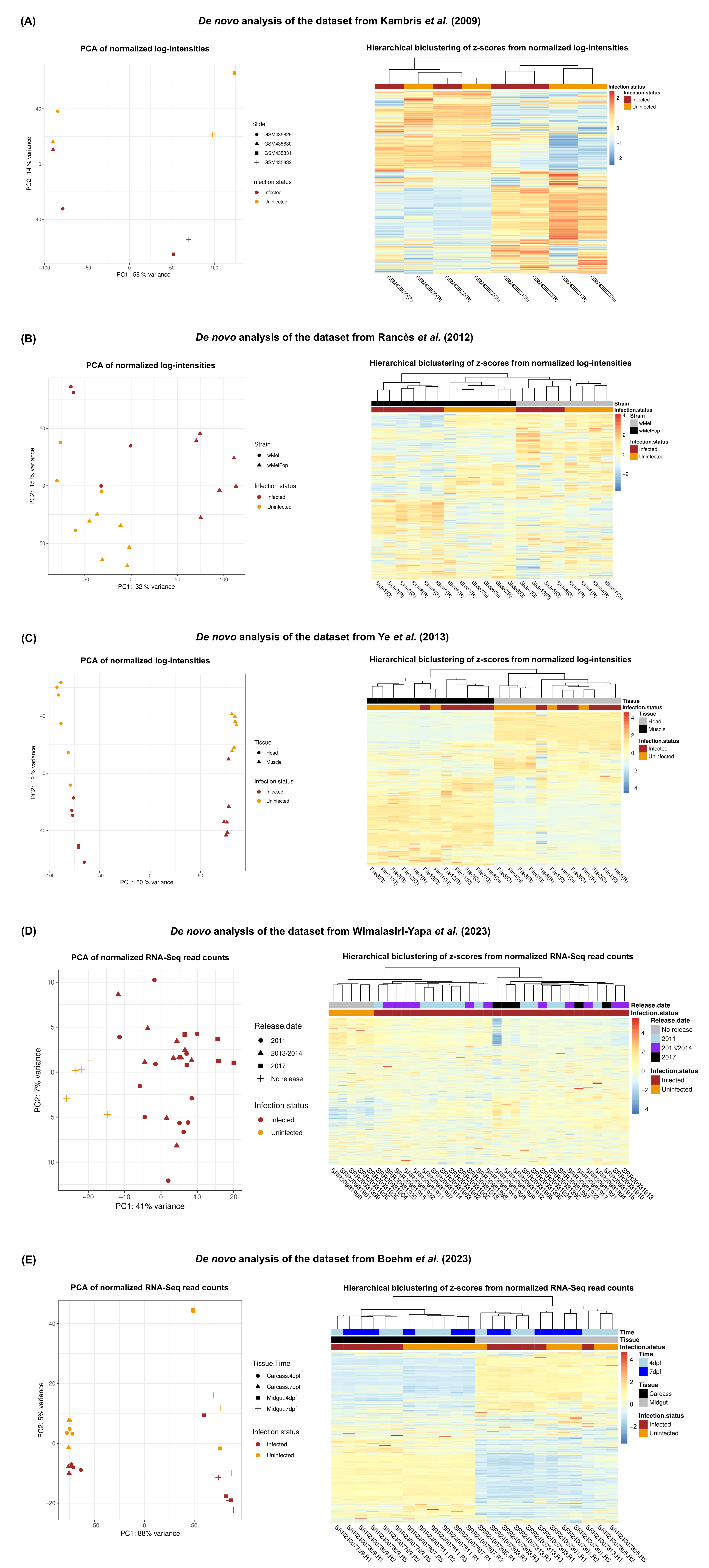

Supplement: Supplementary Figure 1 — Microarray MA-plots. MA-plots of two-channel microarray data from (A) Kambris et al. (2009), (B) Rancès et al. (2012) and (C) Ye et al. (2013). MA-plots show M values (logarithmic fold-change between red and green intensities for each spot) versus A values (average of logarithmic red and green intensities for each spot). Control spots are colored (see Legend). [file Image1.tif]

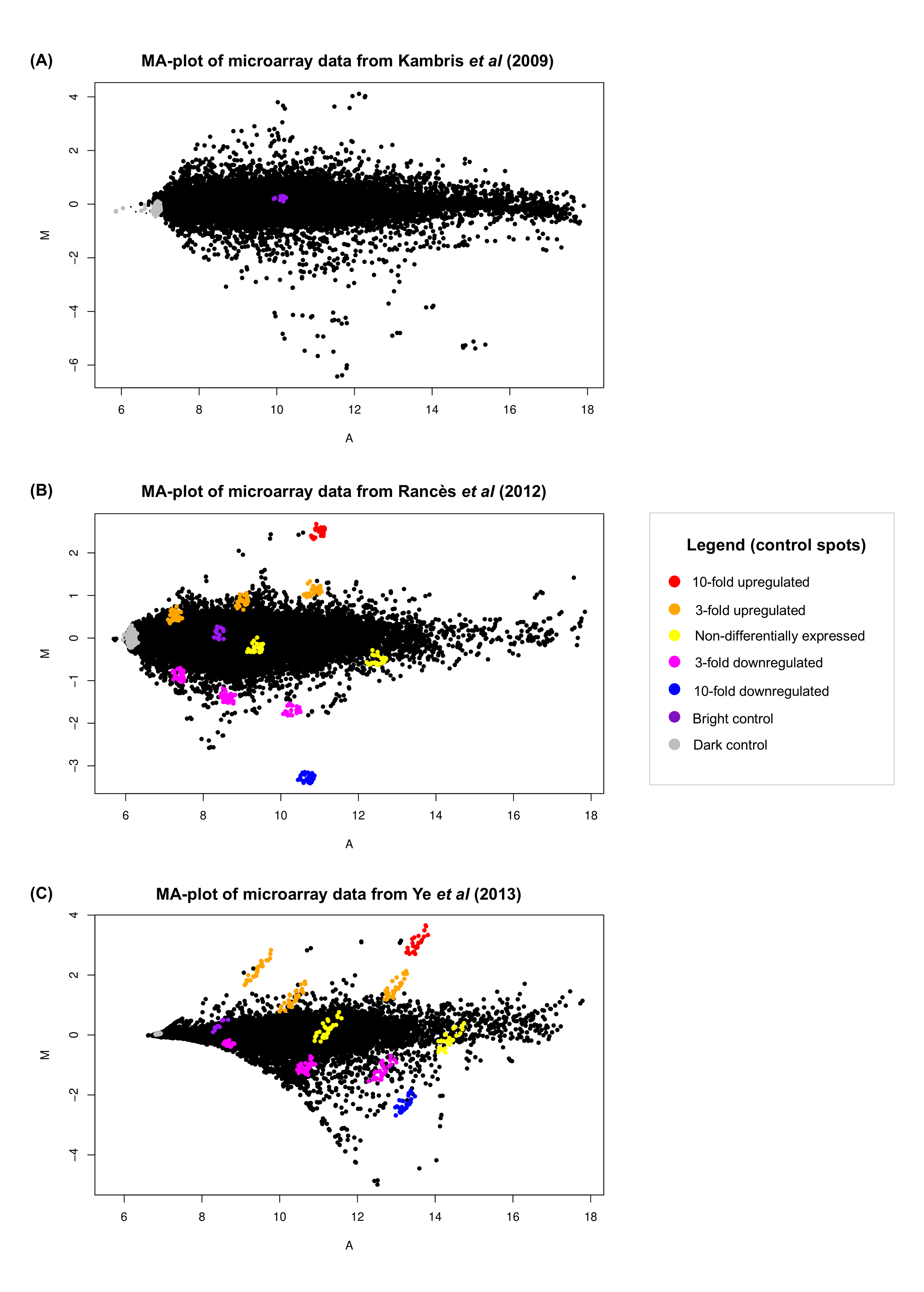

Supplement: Supplementary Figure 2 — Principal component analysis and hierarchical biclustering of transcriptomic datasets. De novo analysis of datasets from (A) Kambris et al. (2009), (B) Rancès et al. (2012), (C) Ye et al. (2013), (D) Wimalasiri-Yapa et al. (2023) and (E) Boehm et al. (2023). [file Image2.tif]
